# Fasting insulin, insulin resistance, and risk of cardiovascular or all-cause mortality in non-diabetic adults: a meta-analysis

**DOI:** 10.1042/BSR20170947

**Published:** 2017-09-07

**Authors:** Xiaohong Zhang, Jun Li, Shuiping Zheng, Qiuyun Luo, Chunmei Zhou, Chaoyang Wang

**Affiliations:** 1 Department of Cardiovascular Diseases, Jingmen Traditional Chinese Medical Hospital (Jingmen Shihua Hospital), Jingmen 448000, China; 2 Department of Endocrinology, Jingmen Traditional Chinese Medical Hospital (Jingmen Shihua Hospital), Jingmen 448000, China

**Keywords:** all-cause mortality, cardiovascular mortality, HOMA-IR, insulin, insulin resistance, meta-analysis

## Abstract

Studies on elevated fasting insulin or insulin resistance (IR) and cardiovascular or all-cause mortality risk in non-diabetic individuals have yielded conflicting results. This meta-analysis aimed to evaluate the association of elevated fasting insulin levels or IR as defined by homeostasis model assessment of IR (HOMA-IR) with cardiovascular or all-cause mortality in non-diabetic adults. We searched for relevant studies in PubMed and Emabse databases until November 2016. Only prospective observational studies investigating the association of elevated fasting insulin levels or HOMA-IR with cardiovascular or all-cause mortality risk in non-diabetic adults were included. Risk ratio (RR) with its 95% confidence intervals (CIs) was pooled for the highest compared with the lowest category of fasting insulin levels or HOMA-IR. Seven articles involving 26976 non-diabetic adults were included. The pooled, adjusted RR of all-cause mortality comparing the highest with the lowest category was 1.13 (95% CI: 1.00–1.27; *P*=0.058) for fasting insulin levels and 1.34 (95% CI: 1.11–1.62; *P*=0.002) for HOMA-IR, respectively. When comparing the highest with the lowest category, the pooled adjusted RR of cardiovascular mortality was 2.11 (95% CI: 1.01–4.41; *P*=0.048) for HOMA-IR in two studies and 1.40 (95% CI: 0.49–3.96; *P*=0.526) for fasting insulin levels in one study. IR as measured by HOMA-IR but not fasting insulin appears to be independently associated with greater risk of cardiovascular or all-cause mortality in non-diabetic adults. However, the association of fasting insulin and HOMA-IR with cardiovascular mortality may be unreliable due to the small number of articles included.

## Introduction

Insulin resistance (IR) is defined as the inability of insulin to increase cellular glucose uptake and utilization, leading to compensatory hyperinsulinemia [1]. IR can be measured by a hyperinsulinemic-euglycemic clamp, indirect estimates of homeostasis model assessment (HOMA) or calculated using dynamic oral glucose tolerance test. The hyperinsulinemic-euglycemic clamp method is recognized as the gold standard for estimating IR [2]. However, this procedure is not suitable for large population-based studies due to its invasive and time-consuming nature. HOMA-IR estimating IR from fasting glucose and insulin levels is particularly appropriate for large epidemiological studies [3]. There is good correlation between values of IR obtained by HOMA-IR and the hyperinsulinemic-euglycemic clamp procedure [4].

IR promotes the development of atherosclerosis through increasing insulin and glucose levels. Hyperinsulinemia and hyperglycemia can exert direct atherogenic effect on the vessel wall [5]. IR also reduces the ability of adipose tissue to store proatherogenic lipids and produces a variety of proinflammatory mediators from the adipose tissue [6]. All these factors thus contribute to atherosclerosis.

Hyperinsulinemia or HOMA-IR has been considered as a surrogate measure of IR [7]. Several prospective studies [8–10] but not all [11–15] have shown that hyperinsulinemia or HOMA-IR was associated with greater risk of cardiovascular or all-cause mortality. A previous meta-analysis [16] suggested that hyperinsulinemia was independently associated with an exacerbated risk of cardiovascular mortality in non-diabetic individuals. Another meta-analysis [17] indicated that elevated fasting insulin and HOMA-IR were associated with higher risk of cardiovascular disease in non-diabetic individuals. However, these two well-designed meta-analyses did not evaluate the association of fasting insulin or HOMA-IR with all-cause mortality risk.

This meta-analysis aimed to evaluate the association of elevated fasting insulin/HOMA-IR with cardiovascular or all-cause mortality risk in non-diabetic adults on the basis of prospective observational studies.

## Materials and methods

### Search strategy

The present study was performed in accordance with the checklist of Meta-analysis of Observational Studies in Epidemiology reporting guidelines [18]. Two authors (X. Zhang and J. Li) independently made the literature search on PubMed and Embase databases until November 2016 without language restrictions. The following keywords in various combinations were used: ‘insulin’ OR ‘hyperinsulinemia’ OR ‘insulin resistance’ OR ‘homeostasis model assessment’ OR ‘HOMA-IR’ AND ‘mortality’ OR ‘death’ AND ‘prospective’ OR ‘longitudinal’ OR ‘follow-up’. We also manually searched the reference list of the included studies to identify any additional eligible articles.

### Study selection

The following inclusion criteria were applied: (i) prospective observational studies enrolling non-diabetic adults; (ii) fasting insulin levels or IR measured by HOMA as exposure; and (iii) reported multiple adjusted risk ratio (RR) or hazard ratio (HR) with 95% confidence interval (CI) of cardiovascular or all-cause mortality comparing the highest with the lowest category of fasting insulin or HOMA-IR. Studies were excluded if: (i) enrollment of patients with diabetes at baseline; (ii) unavailable fasting insulin or HOMA-IR data; (iii) reported risk estimate by continuous value of fasting insulin levels or HOMA-IR. Data from the cross-sectional, case–control, or retrospective studies, reviews, and conference abstract were also excluded.

### Data extraction and quality assessment

Two authors (X. Zhang and J. Li) independently extracted the data and assessed the study quality from the included studies. Any disagreements in these phases were resolved by consensus. The extracted data included: first author’s surname, publication year, country of origin, study design, number of participants, proportion of women, age at baseline, fasting time, methods of insulin assay, formula of HOMA-IR calculation, cut-off value of fasting insulin or HOMA-IR comparison, total number of death events, fully adjusted risk estimate, duration of follow-up, and adjustment of variables. Study quality was assessed by a 9-star Newcastle–Ottawa Scale (NOS) for cohort studies†. NOS stars of 7 or more were considered as high-quality studies.

### Statistical analyses

All statistical analyses were performed using STATA software (version 12.0, Stata, College Station, TX, U.S.A.). HR were assumed to approximate the same measure of RR. We considered the presence of significant heterogeneity across studies according to *P*<0.1 for Cochran *Q*-statistic and quantitated by the *I*^2^ tests with its value ≥50%. A random-effects model was chosen in the presence of significant heterogeneity; otherwise, a fixed-effect model was selected‡. Publication bias was planned by an assessment of the Begg’s test [19] and Egger’s test [20] if more than ten articles were retrieved. Sensitivity analyses were conducted by removing a single study from the overall analysis at each turn.

## Results

### Search results and study characteristics

**Figure 1:**
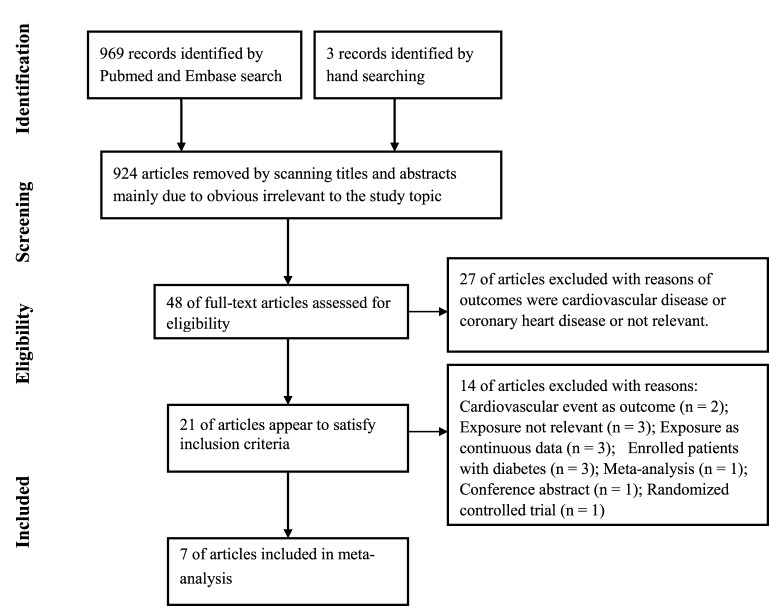
Flow chart of the study selection process

Figure 1 depicts the detailed process of the study selection**.** A total of seven studies [8–13,15] were included in the final meta-analysis. Table 1 summarizes the baseline characteristics of the included studies. Seven articles involving 26976 non-diabetic individuals were identified. Sample sizes ranged from 743 to 6074 in the individual studies. Three articles [9,13,15] were conducted in the U.S.A., three [8,11,12] in Europe, and one [10] in Korea. Two articles [11,12] consisted of men only. Three articles [11–13] only reported fasting insulin levels, three articles [8,10,15] reported HOMA-IR only, and one article [9] simultaneously reported fasting insulin and HOMA-IR. The follow-up duration ranged from 5.0 to 19.0 years. Using the NOS scale, the overall NOS in each study ranged from 6 to 8 stars (Supplementary Table S1).

**Table 1:** Baseline characteristics of the selected studies

Study (year)	Study region	Design	Subjects (% female)	Age (years)	Fasting times	Insulin assay	Insulin comparison	IR definition	HOMA-IR comparison	RR/HR (95% CI)	Follow- up (years)	Adjusted for variables
Lakka et al. (2000) [11]	Finland	Prospective population- based study	1521 (0)	42–60	12 h	RIA	Quintile (pmol/L) 4 compared with 1; ≥90 compared with <52	–	–	CV death: 45; 1.4 (0.5–4.0)	9.5	Age, examination years, smoking, alcohol, HDL, TG, BMI, SBP, DBP, WC, blood leukocyte count, apolipoprotein B, fibronogen, and maximal oxygen uptake
Hedblad et al. (2002) [8]	Sweden	Prospective cohort study	4748 (60.8)	46–68	Overnight	RIA	–	Fasting insulin (μU/ml) × glucose (mmol/l)/ 22.5	1.80 for women and 2.12 for men	Total deaths: 93; 1.62 (1.03–2.55)	5	Age, sex, SBP, hypertension, HDL, TG, glucose, WC, smoking, and leisure-time PA.
Nilsson et al. (2003) [12]	Sweden	Prospective cohort	6074 (0)	25–63	Not reported	RIA	Tenth decentile compared with others; 21–140 compared with 1–20 mU/l	–	–	Total deaths: 1012; 1.17 (0.96–1.41)	19	Age, SBP, TC, TG, smoking, fasting glucose, and BMI
Ausk et al. (2010) [9]	U.S.A.	Prospective cohort study	5511 (NP)	≥20	10–16 h for morning test or 6 h for other tests	RIA	Quintile (pmol/l) 4 compared with 1; >11.5 compared with ≤6.15	Fasting insulin (μU/ml) × glucose (mmol/l)/ 22.5	Quintile 4 compared with quintile 1; >2.8 compared with ≤.4	Total deaths: 643; 1.64 (1.1–2.5) IR; 1.48 (0.9–2.2) insulin; CV deaths: 237; 3.2 (1.7–5.9) IR	8.5	Age, sex, BMI, waist-to-hip ratio, race/ethnicity, smoking, alcohol, PA, SBP, DBP, TC, HDL, TG, education, and CRP
de Boer et al. (2012) [13]	U.S.A.	Prospective, community- based cohort	3138 (61)	72 ± 5.0	Not reported	RIA	Quintile (IU/ml) 4 compared with 1; >18 compared with <10	—	—	Total deaths: 1810; 1.05 (0.89–1.25)	14.7	Age, sex, race, study site, prevalent CVD, smoking, lipid-lowering medication, LDL, PA, WC, SBP, DBP, antihypertensive drugs, HDL, triglyceride, CRP, and eGFR
Kim et al. (2013) [10]	Korea	Prospective community- based study	743 (57.5)	76.4 ± 9.3	≥12 h	RIA	–	Fasting glucose (mg/l) × insulin (μU/ml)/405	Quintile 5 compared with quintile 3; >1.5 compared with 0.85–1.07	Total deaths: 168; 2.01 (1.06–3.90);	5.2	Age, TC, hemoglobin, eGFR, proteinuria, CRP, and DBP
Kim et al. (2015) [15]	U.S.A.	Prospective study	5241 (56.2)	58.9 ± 13.9	≥8 h	RIA and immunoenzymometric assay	–	Fasting insulin (μU/ml) × glucose (mmol/l)/ 22.5	Quintile 4 compared with quintile 1; >2.67 compared with <1.29	Total deaths: 724; 1.1 (0.9–1.5); CV deaths: 316; 1.5 (1.0–2.2)	6.6	Age, gender, race/ethnicity, smoking, survey cycle, BMI, LDL, HDL, and SBP

Abbreviations: BMI, body mass index; CBD, cerebrovascular disease; CHD, coronary heart disease; CRP, C-reactive protein; CV, cardiovascular; CVD, cardiovascular disease; DBP, diastolic blood pressure; eGFR, estimated glomerular filtration rate; HDL, high-density lipoprotein; LDL, low-density lipoprotein; NP, not provided; PA, physical activity; PAD, peripheral vascular disease; SBP, systolic blood pressure; TC, total cholesterol; TG, triglyceride; WC, waist circumference.

### Association of fasting insulin and HOMA-IR with all-cause mortality

**Figure 2:**
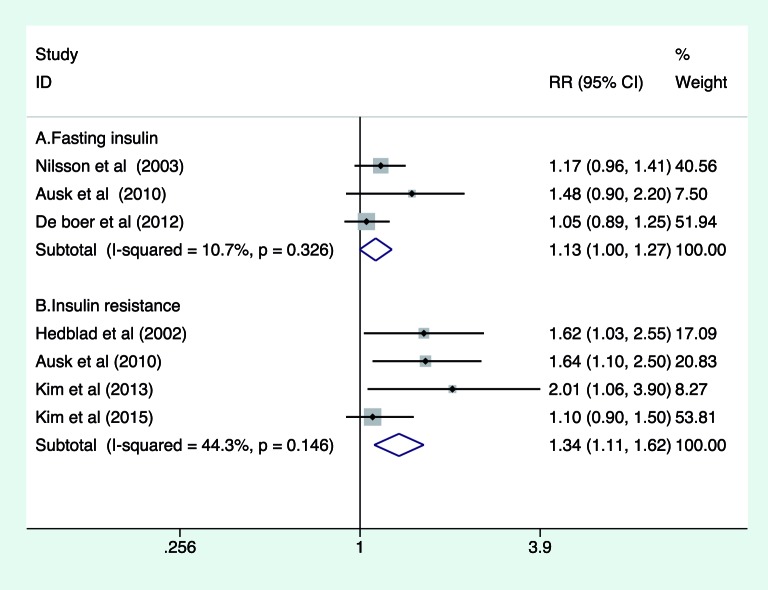
Forest plots showing pooled RR and 95% CI of all-cause mortality for the highest compared with lowest category of fasting insulin level and IR

Three studies [9,12,13] reported the association of fasting insulin levels with all-cause mortality risk. As shown in Figure 2(A), the pooled RR of all-cause mortality was 1.13 (95% CI: 1.00–1.27; *P*=0.058) for the highest compared with lowest category of fasting insulin levels in a fixed-effect model. There was no evidence of heterogeneity across studies (*I*^2^ =10.7%, *P*=0.326). Sensitivity analyses indicated that there were slight changes in magnitude of the combined risk estimate when any single study was excluded.

Four studies [8–10,15] reported the association of HOMA-IR with all-cause mortality risk. As shown in Figure 2(B), the pooled RR of all-cause mortality was 1.34 (95% CI: 1.11–1.62; *P*=0.002) for the highest compared with lowest category of HOMA-IR in a fixed-effect model, with no evidence of significant heterogeneity (*I*^2^ =44.3%, *P*=0.146). Sensitivity analysis by removing individual study at a time showed that there were slight changes in magnitude of the pooled risk summary (results not shown).

### Association of fasting insulin and HOMA-IR with cardiovascular mortality

**Figure 3:**
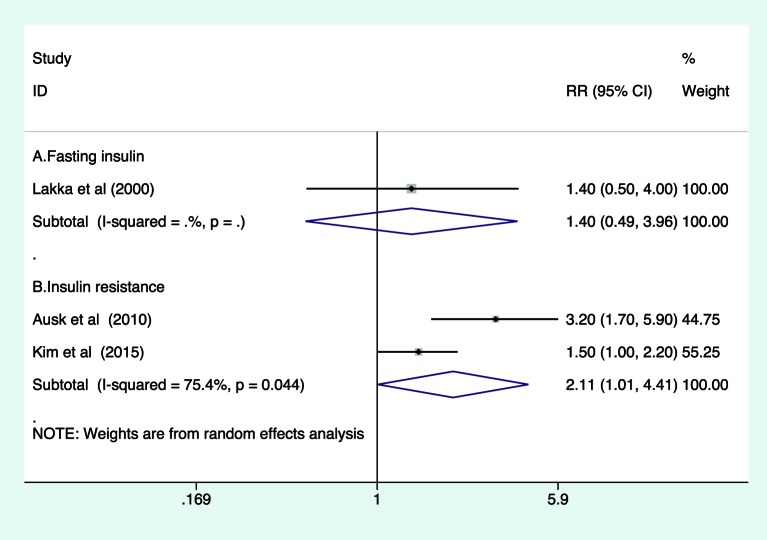
Forest plots showing pooled RR and 95% CI of cardiovascular mortality for the highest compared with lowest category of fasting insulin level and IR

As shown in Figure 3, the pooled RR of cardiovascular mortality for the highest compared with lowest category was 1.40 (95% CI: 0.49–3.96; *P*=0.526) for fasting insulin levels in one study [11] and 2.11 (95% CI: 1.01–4.41; *P*=0.048) for HOMA-IR in two studies [9,15] in a random-effect model. There was evidence of significant heterogeneity between two studies (*I*^2^ =75.4%, *P*=0.044).

### Subgroup analyses and publication bias

As for the small number of studies included, we did not conduct subgroup analysis and publication bias because statistical tests for these analyses may be potentially unreliable.

## Discussion

This is the first meta-analysis to investigate the association of fasting insulin levels and IR with all-cause mortality risk in non-diabetic adults. Our meta-analyses suggested that IR as measured by HOMA-IR appeared to be independently associated with greater risk of cardiovascular and all-cause mortality. When quantitated as the highest compared with lowest category, individuals with the highest HOMA-IR had a 111 and 34% greater risk of cardiovascular and all-cause mortality, respectively.

IR is considered a better biomarker than insulin or glucose alone because it incorporates both the biomarkers. Our meta-analysis indicated that HOMA was associated with greater risk of all-cause mortality in adults without diabetes; however, the predictive role of elevated fasting insulin itself in this process was not statistically significant. These findings should be taken with caution because the result was established on an indirect comparison. We could not directly compare the magnitude of the association of HOMA-IR and fasting insulin because most studies failed to report results simultaneously.

A study-level meta-analysis [16] of prospective data from 11 DECODE study populations suggested that both fasting insulin and HOMA-IR were independently associated with cardiovascular mortality in non-diabetic men and women. However, this well-designed meta-analysis did not evaluate the effects of fasting insulin and HOMA-IR on all-cause mortality risk. Our meta-analysis particularly focussed on the association of fasting insulin and HOMA-IR with all-cause mortality risk. We found that only IR as measured by HOMA-IR was associated with higher risk of all-cause mortality in non-diabetic adults.

Mechanisms underlying IR on the development of cardiovascular and all-cause mortality are considered to be through the direct atherogenic action of insulin on vessel wall [5,6] and/or indirect through obesity, blood pressure, lipids and metabolic homeostasis [21]. IR is the core metabolic abnormality in metabolic syndrome. Nevertheless, metabolic syndrome was an important risk factor for all-cause mortality [22]. Moreover, IR has been associated with type 2 diabetes [23], hypertension [19], cardiovascular disease [20], and a variety of cancers [24,25]. All above-mentioned diseases contribute to the risk of premature mortality.

This meta-analysis has several limitations. First, HOMA-IR strongly reflects hepatic IR than the total effect of systemic IR [4] and we only included studies using the HOMA-IR index to estimate IR. Second, analyses related only to single baseline value of fasting insulin and HOMA-IR; therefore, misclassification of individuals in each category could not be excluded. Time average insulin and HOMA-IR analysis in future studies will further confirm the observed association. Third, we could not determine the optimal cut-off value of HOMA-IR due to different formulas of HOMA-IR used. Finally, our meta-analysis was a study-level meta-analysis not a individual-level meta-analysis, therefore, we cannot control some potential confounders. Lack of adjustment for dietary patterns and degrees of physical activity, prediabetes, obesity, physical activity, renal function, or medications may lead to slight overestimation of the risk estimates.

In summary, this meta-analysis suggests that IR as measured by HOMA-IR appears to be independently associated with greater risk of all-cause mortality in non-diabetic adults. Early ascertaining of IR may help to improve all-cause mortality risk stratification amongst the non-diabetic adults. However, the association of fasting insulin and HOMA-IR with cardiovascular mortality may be unreliable due to the small number of articles included. More well-designed prospective studies are needed to confirm the findings of this meta-analysis.

## Supporting information

**Supplemental Table S1:** Quality assessment of studies included in the meta-analysis

## Footnotes

† Wells, G., Shea, B., O’Connell, D., Peterson, J., Welch, V., Losos, M. et al. The Newcastle–Ottawa Scale (NOS) for assessing the quality of nonrandomized studies in meta-analyses. http://www.ohri.ca/programs/clinical_epidemiology/oxford.asp (Accessed 3 September 2017).

‡ Higgins, J.P.T. and Green, S.E. Cochrane Handbook for Systematic Reviews of Interventions. Version 5.1.0 [updated March 2011], The Cochrane Collaboration http://training.cochrane.org/handbook (Accessed 3 September 2017).

## Abbreviations

CI: confidence interval
HOMA: homeostasis model assessment
HOMA-IR: homeostasis model assessment of insulin resistance
HR: hazard ratio
IR: insulin resistance
NOS: Newcastle–Ottawa Scale
RR: risk ratio
